# Single-cell transcriptomics reveals zinc and copper ions homeostasis in epicardial adipose tissue of heart failure

**DOI:** 10.7150/ijbs.82844

**Published:** 2023-07-31

**Authors:** Rongjun Zou, Miao Zhang, Zengxiao Zou, Wanting Shi, Songtao Tan, Chaojie Wang, Wenliu Xu, Jiaqi Jin, Stephen Milton, Yang Chen, Ge Wang, Xiaoping Fan

**Affiliations:** 1 The Second Clinical College of Guangzhou University of Chinese Medicine, Guangzhou 510405, Guangdong, China.; 2 Department of Cardiovascular Surgery, Guangdong Provincial Hospital of Chinese Medicine, the Second Affiliated Hospital of Guangzhou University of Chinese Medicine, Guangzhou 510120, Guangdong, China.; 3 State Key Laboratory of Dampness Syndrome of Chinese Medicine, Guangzhou 510120, Guangdong, China.; 4 Guangzhou Women and Children's Medical Center, Guangzhou Medical University, Guangzhou, 510623, Guangdong, China.; 5 School of Pharmaceutical Sciences, Key Laboratory of Chinese Medicinal Formulae, Guangzhou University of Chinese Medicine, Guangzhou 510405, China; 6 School of Pharmaceutical Sciences, Stanford University, 450 Serra Mall, Stanford, CA 94305, USA.

**Keywords:** single-cell RNA sequencing, epicardial adipose tissue, development, heart failures, immune microenvironment

## Abstract

Epicardial adipose tissue (EAT) is a unique visceral fat reservoir that shares an immune microenvironment without a distinct boundary with myocardium. Increasingly, visceral fat has been studied as a secondary immune organ, and EAT is no exception in this regard. Cellular subsets of EAT are associated with disease development. In heart failure (HF) patients, however, the immune characteristics of EAT have rarely been studied, especially those non-immune cells related to the immune microenvironment. Herein, an analysis of seven EAT samples by single-cell RNA sequencing (scRNA-Seq) is presented here, including 1 neonate, 1 infant, 1 child, 2 adults with heart failure (Adults-HF) and 2 adult heart transplant donors as non-heart failure control (Adults-Non HF). Analysis of 51730 high-quality cells revealed eleven major cell types in EAT. For the first time, the pseudo-temporal reconstruction technique was employed to plot the cell trajectories of various major cell types (such as T lymphocytes, fibroblasts, endothelial cells, monocytes, and smooth muscle cells) in EAT across different developmental stages, achieving a single-cell resolution. The dynamic gene expression patterns of major cell types presented the immune characteristics of metabolism disorder of zinc and copper ions, and downregulated immune-related pathways in EAT of adult patients with HF. These data provide insights regarding HF immune dysregulation at the cellular level.

## Introduction

EAT is a metabolically dynamic depot of visceral fat situated in the interstitial space between the myocardium and the pericardium, predominantly comprising adipocytes^1^. In addition to adipocytes, EAT also contains immune cells, nerve cells, endothelial cells, vascular cells, etc.^2^. EAT is not only a complex endocrine organ, but also shares the coronary microcirculation with myocardium without fascial interruption and may significantly affect cardiac function^3^. Cardiovascular diseases are mediated by immune and non-immune cells of EAT, and EAT has received increasing attention and been widely studied as a secondary immune organ in recent years. At present, little research have described the associations between non-immune cells in EAT and the etiology of cardiovascular disorders. For example, endothelial cells and vascular smooth muscle cells (VSMCs) in EAT are involved in the occurrence and development of coronary atherosclerosis^4^.

EAT is populated by immune cells including adaptive immune cells (especially T and B lymphocytes) and innate immune cells (NK cells, macrophages, dendritic cells etc.). Macrophages are the most prevalent immune cell population in visceral fat^5^. In a study conducted by Yoichiro Hirata et al., it was discovered that the ratio of M1/M2 macrophages in EAT was exhibited a positive correlation with the clinical severity of coronary artery disease (CAD)^6^. T lymphocytes, which are integral to the adaptive immune system, were found to be the second most abundant immune cell type. Immunohistochemical analysis revealed the accumulation of CD8+ T cells in the EAT of CAD patients^7^, and similar accumulation was observed in patients undergoing on-pump coronary artery bypass grafting^8^. Few studies have focused on the other immune cells in EAT. Srikakulapu et al. suggested that the B1 cells in perivascular adipose tissue (PVAT) have the potential to protect the aorta and coronary arteries^9^. Mraz et al. demonstrated that CAD was accompanied by an increased amount of T and B lymphocytes, while natural killer (NK) cells were found to be reduced in human EAT^10^.

As mentioned above, the existing researches mainly pay attention on the impact of immune cells in EAT on the pathogenesis of diseases, and the changes in immune cells of EAT during growth and development stages are still unclear. Shalini Ojha et al. illustrated significant changes in the histological and transcriptome characteristics of EAT during the transition period from neonatal to childhood. The expressions of EAT microarray genes in infants (1-12 months old) were similar to those in children (2-7 years old), but significantly different from neonates (0-24 days of age)^11^. Whether this difference exists in the diverse types of cells of EAT during the development stages? In this paper, the immune functional changes of different cell types in EAT of neonates, infants, and adults were investigated through pseudo-time analysis of single-cell transcriptome sequencing data.

Heart failure is characterized by a disruption in cardiac circulation, resulting from venous blood stasis and inadequate arterial perfusion due to impaired systolic and/or diastolic function^12^. HF is a prominent healthcare concern that affects a substantial number of individuals globally, with an estimated prevalence of 1% to 2% among adults in developed nations^13^. Left ventricular ejection fraction (LVEF) was generally regarded as a clinically phenotypic marker in patients with HF^14^. Nevertheless, the utilization of LVEF in clinical trials has resulted in an oversimplified understanding of this intricate syndrome^15^.

Emerging evidence suggests that the total volume of EAT plays a significant role in HF, especially among patients with preserved ejection fraction (HFpEF), as compared to healthy individuals^16^. EAT can impact cardiac function in HF patients through various mechanisms, including inflammation, immune and autonomic disorders, and the mechanical effects of fibrotic fat pads. However, under pathological conditions, especially in HFpEF patients^17^, EAT undergoes a pro-inflammatory transformation and becomes detrimental to cardiac health. It has been reported that EAT in HF patients was exhibits pronounced immune activation, especially the activation of T lymphocytes. Further analysis revealed a significant amplification of T lymphocytes in EAT, with a dominance of IFN-γ+ effector memory T lymphocytes (Tem)^18^.

In addition, Adamo et al. found that the bulk myocardial B cells remain within the intravascular space, with only a small fraction crossing the endothelium into myocardial tissue^19^. Horckmans et al. detected major clusters of B cells in EAT of patients with CAD in contrast to CAD-free controls^20^. Butcovan et al. revealed the correlation between the decreased EAT thickness and the increased infiltration of macrophages in cardiac patients with chronic cardiac failure^21^. Since the changes of various cellular immune functions in EAT are closely linked to the genesis and progression of HF, a reasonable hypothesis was proposed to reduce the risk of HF by improving microenvironment of immune cells within EAT.

Hence, we attempted to clarify the developmental trajectories of epicardial fat immune cells at different developmental stages, and find critical markers of the developmental differentiation nodes of EAT immune cells using single-cell transcriptome sequencing technology. In addition, the heterogeneity of EAT immune cells in HF patients and non-HF patients was compared to search for markers of transcriptome differences, providing new ideas for the treatment of HF patients.

## Materials and Methods

### Clinical sample collection

The study subjects for scRNA-seq were divided into five cohorts with a total of seven participants, including one neonate, one infant, one child, 2 Adults-HF, and 2 Adults-Non HF (see Supplementary material online, Table S1). Fresh EAT samples of these seven subjects were surgically excised and immediately soaked in GEXSCOPE tissue preservation solution at 4 °C for later preparation of single-cell suspensions. This investigation received ethical approval from the Research Ethics Review Committee of the Second Affiliated Hospital of Guangdong Hospital of Traditional Chinese Medicine (approval number: ZF2023-136). Prior to sample collection, informed consent was duly acquired from all participants.

### Cell dissociation, cDNA library preparation and Single-cell RNA sequencing

Fresh EAT samples were rinsed over ice with phosphate buffer saline (PBS) and then chopped into small pieces (1-2 mm^3^). EAT cells were isolated using digestive enzymes (including 3 mg/mL type I collagenase, 0.156 mg/mL type XI collagenase, and 60 U/mL DNase I) in 37°C water bath for 45min. Digestion was terminated through the addition of Dulbecco Modified Eagle Medium (DMEM) supplemented with 10% fetal bovine serum. The cells were filtered out from the cell-enzyme mixture using a 40-µM stainless nylon mesh (Corning Falcon, USA). The filtrate was centrifuged at 300 ×g and 4 °C for 5 min, and cell sediment was resuspended in 1X PBS on ice for single-cell capture. The concentration and activity of cells were measured using a TC20 automatic cell counter (Singleron) following Trypan blue (302643, Sigma) staining. Cells with > 80% viability and concentration > 1×10^6^ cells/mL were used for subsequent library preparation.

Single-cell was captured by loading the qualified EAT cell suspension of each sample on a Chromium Controller (10× Genomics, California, USA) in accordance with the standard protocol. cDNA synthesis and library preparation were conducted following the instructions provided in the Single Cell 3′ Reagent Kits User Guide Version 3.1 (10× Genomics, California, USA). Finally, the libraries subjected to sequencing on an Illumina HiSeq X Ten system. The primary genomic information outlined in this study has been securely stored in the Genome Sequence Archive (GSA)^22^, which is housed within the esteemed National Genomics Data Center (NGDC)^23^. The deposited data, with the accession codes GSA-Human: HRA004960, are readily accessible to the public via the following web address: https://ngdc.cncb.ac.cn/gsa-human.

### Processing of single-cell sequencing data

The original FASTQ files were aligned with the human GRCh38 reference genome employing CellRanger (version 4.0.0), and unique molecular identifier (UMI) gene expression profiles were generated for each single cell using 'cellranger count' function. The 'cellranger aggr' function was used to normalize gene expression counts that generated from 'cellranger count' to the same sequencing depth to eliminate deviation, and the characteristic barcoded single-cell gene expression matrix was recalculated and generated. High-quality cells with > 700 and < 15000 unique genes, and less than 25% mitochondrial genome transcripts were reserved for downstream bioinformatic analysis. Genes that were expressed in less than eleven cells were deleted for further analysis to avoid a decrease in average cell expression. To identify and eliminate doublets within a range of 7.5% in the original dataset, DoubletFinder algorithm (https://github.com/chris-mcginnis-ucsf/DoubletFinder) was employed.

The 'NormalizeData' function in Seurat software (https://satijalab.org/seurat/pbmc3k_tutorial.html) was used for normalization with default settings. Thereafter, the final high-quality single-cell gene expression matrix was generated after eliminating cell cycle and mitochondrial effects by Seurat package.

### Identification of the major Cell types and their subtypes

The highly variable genes (HVGs) were selected from the final high-quality count matrix via the 'FindVariableGenes' function, and the dimension reduction algorithm of principal component analysis (PCA) was performed on the datasets using the 'RunPCA' function in Seurat with default parameters. The identification of major cell clusters was stem from average gene expression of their typical markers using the 'FindClusters' function, and the clustering parameter resolution was set to 0.8. To explore the transcriptional changes in subsets of several target cell types, the sub-clusters was identified using the same functions as described above and the clustering parameter resolution was set to 0.6. Subsequently, two-dimensional visualization of the clustered and sub-clustered cells was performed using Uniform Manifold Approximation and Projection (UMAP) method (https://satijalab.org/seurat/articles/get_started.html) by setting dimension parameters to 1:30.

'FindAllMarkers' function (test.use=bimod) in Seurat was used to screen marker genes for each cell cluster by comparing all gene expression files of one cluster to those of all other clusters with the default bilateral nonparametric Wilcoxon rank sum test. Then, marker genes were identified based on the functions of differentially expressed genes (DEGs) in different cell cluster, the known biological roles of the marker genes, and the distance relationships between cells in UMAP maps. In this study, the major cell types were manually annotated according to classical marker genes and their known biological functions.

To further detect marker genes for each sub-cluster of the target major cell types, 'FindAllMarkers' function was also used to compare all gene expression files of one sub-cluster to those of all other sub-clusters. Marker genes were selected from genes with an adjusted *P* value less than 0.05 after regulation by Bonferrin, the mean natural logarithmic of the fold change in expression was at least 0.25, and those expressed greater than 10% in the corresponding sub-clusters. The top 50 genes ranked according to the average fold change of expression were selected as marker genes for the corresponding cell sub-cluster. Seurat-Do-Heatmap/ dot-plot /Vlnplot functions were used to generate heat maps, dot plots, and violin plots of the cell-specific marker genes of each cell type or subtype.

### Differentially expressed gene detection and functional enrichment analysis

DEGs between different conditions (such as between cohorts of Adults-HF and Adults-Non HF) were identified using Wilcoxon rank sum test with Bonferroni correction by the 'FindMarkers' function in Seurat. Genes with adjusted *P* value ≤ 0.05, average log2-fold change (FC) ≥ 0.5 (|log2FC| ≥ 0.5), and detection rate of expressed genes over 10% of the cells within either or both groups were selected as significant DEGs between two groups.

In order to gain insights into the potential functions and mechanisms of the selected genes, such as marker feature genes of different cell types and DEGs between different conditions mentioned above, we conducted Gene Ontology (GO) enrichment analysis. The analysis was performed on the selected genes using the clusterProfiler (v3.14.3) R package^24^. Enrichment fractions (*P*-values) for the GO annotations associated with the selected genes were determined using a hyper-geometrical statistical test, and the false discovery rate (FDR) was estimated basing on Benjamini-Hochberg method. Finally, GO terms with the adjusted *P* value < 0.05 were considered significant. The 'bar plot' function was used for visualization of selected GO terms.

### Gene set variation analysis

Gene set variation analysis (GSVA) 25 was employed in this study to estimate the variability of gene set enrichment results obtained from sequencing. The gene expression matrix was transformed into a pathway expression matrix to evaluate different pathways that enriched in different cell subtypes. In this study, we applied GSVA to pathways related to ribosome synthesis, copper ion metabolism and ubiquitination from several target cell subtypes between different groups (Adults-HF and Adults-Non HF) using GSVA package in R software. The results of the matrix of GSVA enrichment scores were subsequently visualized by heatmap.

### Pseudo-time trajectory construction

Monocle2 R package (v2.14.0) (http://cole-trapnell-lab.github.io/monocle-release/) was applied to deconstruct changes in expression of genes associated with cell state transitions in major cell clusters and sub-clusters. The 'DDRTree' function in Monocle2 R package was utilized to reduce the dimensionality of the datasets, and cells were sorted in a pseudo-temporal order using the 'orderCells' function. The 'reduceDimension' function was applied to infer the Pseudo-time trajectory with default parameters, and the 'plot_cell_trajectory' function was used for visualization of the reconstructed trajectories. The top 50 significantly altered marker genes were extracted from each module, among which were genes clustered based on pseudo-temporal expression patterns, and to generate a representative heat map.

### Cell-cell communication analysis

To explore the potential interactions between various cell types in the microenvironment of HF, we conducted a cell-cell communication analysis using CellPhoneDB (2.1.2)^26^. Normalized counts and annotations of cell subpopulation were utilized as inputs in CellPhoneDB to identify potential ligand-receptor pairs. Ligand-receptor pairs with a communication probability (*P*-value) < 0.05, and the interaction pairs' average expression level greater than 0 were considered significant. The 'dot_plot' function in CellPhoneDB was applied to plot interaction networks of the selected specific pairs.

### RNA volocity analysis

The package scVelo (v0.2.3) was applied to perform RNA velocity analysis using dynamical modeling. The unspliced and spliced messenger RNAs were primarily distinguished in every sample, and then the directional dynamic information was recovered by leveraging RNA-splicing information. Finally, the 'umap' function was used for visualization of the estimated cellular velocities in the UMAP embedding.

## Results

### The cellular landscape of EAT revealed by scRNA-seq

**Figure 1:**
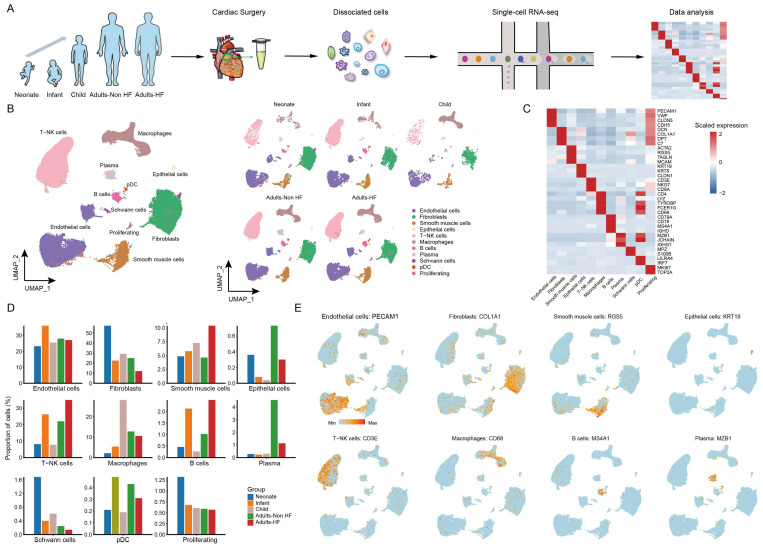
** ScRNA-seq analyses of EAT cells from neonates, infants, children, and adults with and without HF reveal their cellular subpopulations. (A)** Cell sorting and single-cell data analysis workflow. **(B)** The cellular subpopulations within human EAT are visualized through UMAP plots, highlighting both cell identity and different groups. Each dot in the UMAP plots represents a cell, with distinct colors indicating specific cell types. **(C)** A heatmap is presented to display the differentially expressed marker genes across various cell types. The color gradient from blue to red signifies the expression levels, ranging from low to high. **(D)** Histograms of the proportions of each cell type in different groups. **(E)** Feature plots are provided to demonstrate the expression distribution of representative genes for the eight major cell types in EAT, with color-coded expression levels overlaid onto the UMAP plot.

A total of seven EAT samples from five groups (including 1 neonate, 1 infant, 1 child, 2 adults-HF and 2 adults-Non HF) were collected and prepared single-cell suspensions, thereafter were sequenced on the 10× Genomics platform for further bioinformatics analysis (Figure 1A). 51730 high-quality cells were isolated from all EAT samples and retained for downstream analysis after pre-processing and quality filtration. Following gene expression normalization, unsupervised cell clustering algorithm was used to classify all cells in EAT into ten main cell clusters based on their signature genes, including endothelial cells (27.59% of all cells), fibroblasts (25.36%), SMCs (6.92%), epithelial cells (0.42%), T-NK cells (24.13%), myeloid cells (10.47%), B cells (1.52%), plasma (2.06%), Schwann cells (0.46%), and proliferating cells (0.70%) (Figure 1B-C, Supplementary Table S2). The proportions of each cell types in each group are plotted in Figure 1D, and the canonical marker genes for eight major EAT cell types are shown in the feature plots (Figure 1E). As it shown, the proportions of majority cell populations in EAT differed significantly among different groups. The eight major EAT cell types are as follows: endothelial cells (*PECAM1*^high^), fibroblasts (*COL1A1*^high^), SMCs (*RGS5*^high^), epithelial cells (*KRT19*^high^), T-NK cells (*CD3E*^high^), myeloid cells (*CD68*^high^), B cells (*MS4A1*^high^), and plasma (*MZB1*^high^). In addition, the fraction of cells, numbers of cells, UMIs, and genes in each sample are shown in Supplementary Figure S1.

### Functional changes within the T-NK subsets of EAT suggested metabolic disorders of zinc and copper ions in HF patients

**Figure 2:**
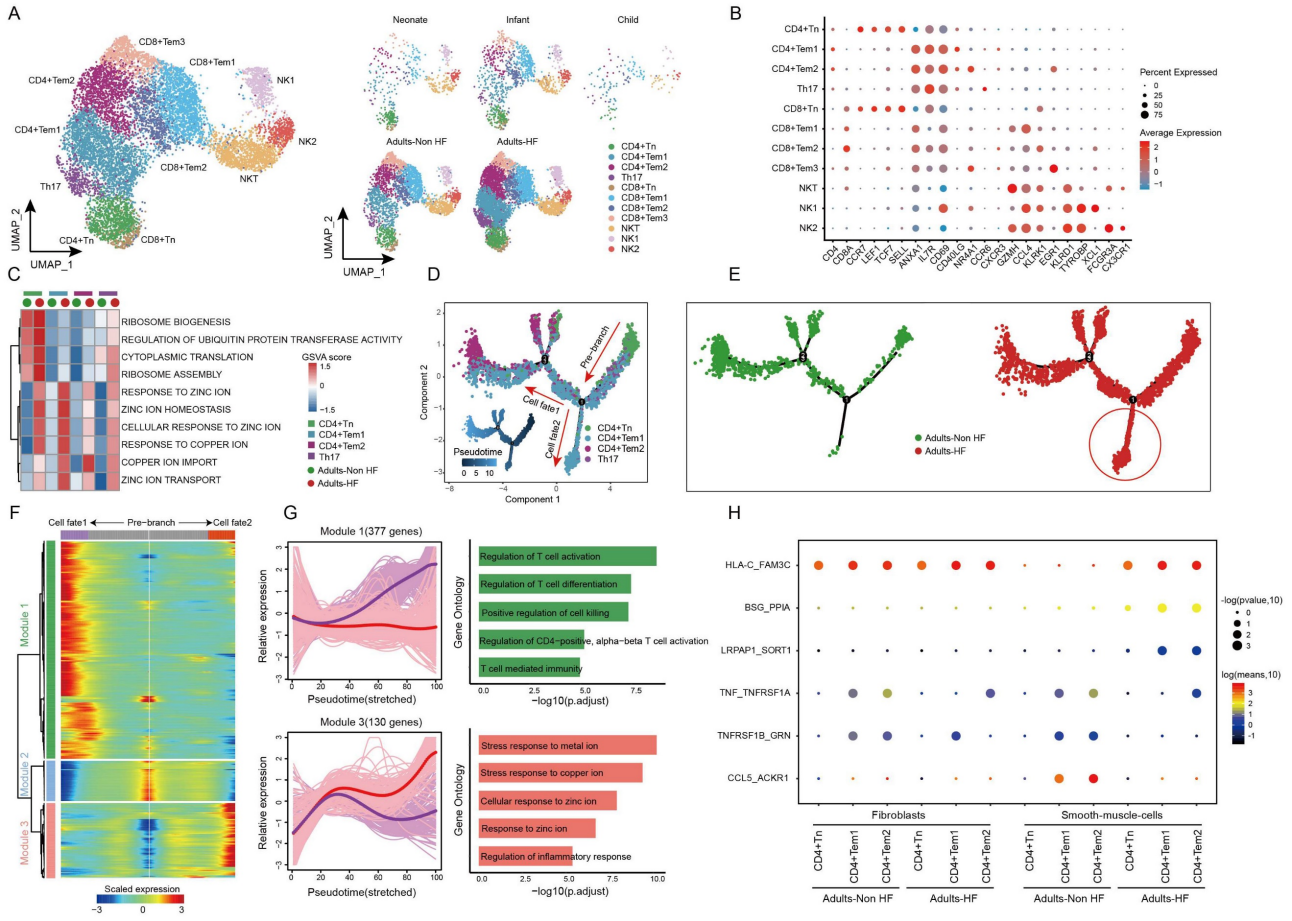
** Diverse subsets were identified in T-NK cells and functional changes suggested metabolic disorders of zinc and copper ions in CD4+ T cells in EAT of HF patients. (A)** UMAP plots visualizing T-NK cell sub-clusters in human EAT based on cell identity (left) and different groups (right). **(B)** Marker genes for T-NK cell subtypes as displayed in a bubble chart. The expression of a gene is marked by the presence of a cercle, the size of which indicates the proportion of cells that expressing this gene. Color gradient from blue to red indicates a low-high level of expression.** (C)** A heatmap shows comparison of pathway activities across different CD4+ cell subtypes in Adults-Non HF and Adults-HF groups by GSVA. Pathway scores are normalized. **(D-E)** Pseudo-time trajectories of the CD4+ T cells inferred by Monocle analysis. Cells are colored based on cell type (D) and group (E).** (F)** Heatmap displaying expression patterns of top DEGs that cataloged into three major modules in a Pseudo-time manner. **(G)** The expression dynamics of genes in modules 1 and 3 under cell fate1 and cell fate 2 states (left), and their enriched biological progresses (right). **(H)** Bubble chart shows selected ligand-receptor interactions between CD4+ T cells and fibroblasts or SMCs.

T and NK cells are important constituents of the immune system, and we further explored the heterogeneity and functional changes among different groups within T-NK cells. Totally, 12484 T-NK cells were detected in EAT, and were divided into 11 subsets based on their highly expressed marker genes: CD4+ naive T cell (CD4+ Tn), CD4+ Tem1, CD4+ Tem2, T helper cell 17 (Th17), CD8+ Tn, CD8+ Tem1, CD8+ Tem2, CD8+ Tem3, Natural killer T cell (NKT), NK1 and NK2 (Figure 2A-B, Supplementary Table S3). The distributions of subsets of T-NK cells in different groups showed a similar trend as the whole T-NK cells (Figure 2B right). The top enriched biological pathways in each CD4+ T cell subtypes were selected and subjected to GSVA analysis between Adults-HF and Adults-Non HF groups. Results showed that the functions of ribosome synthesis and ubiquitination in CD4+ Tn, and the metabolism of zinc ion and copper ion in each CD4+ T cell subtypes were significantly enhanced in Adults-HF group relative to the Adults-Non HF group (Figure 2C).

Using monocle trajectory analysis, we inferred a differentiation trajectory of CD4+ T cells that predominantly originated from the CD4+ Tn cluster and diverged into either the CD4+ Tem1 cluster (cell fate2) or the CD4+ Tem2 cluster (cell fate1) as the terminal differentiation cluster (Figure 2D). The pseudo-time trajectories of CD4+ T cell subsets revealed the significantly higher expressions of genes related to cell fate2 in Adults-HF group compared to that in Adults-Non HF group (Figure 2E). To understand the biological processes driving the pseudo-time components, we examined the co-expression of genes with pseudo-time. As a result, we identified three gene clusters expressed in CD4+ T cells on account of the dynamics of their expression patterns over pseudo-time (Figure 2F). Of note, we observed highly expressed genes in Module 1 (377 genes, eg. *ZNF331*, *GZMH*, *RARA*, *MT-ND3*, *SETX*) associated with T cell activation and differentiation under cell fate1 condition (purple line). Whereas, genes within Module 3 (130 genes, eg.* MT1F*, *MT1M*, *CEMIP2*, *SLC30A1*, *SYNM*), which are involved in metabolisms of copper and zinc ions, were higher expressed under cell fate2 condition (red line) than that under cell fate1 condition (Figure 2G). In other words, the CD4+ T cells in adult patients with HF suffer from metabolic disorders of zinc and copper ions.

To further explore the roles of the CD4+ T cells in the progression of HF, we employed a cell-cell communication analysis to characterize the interactions of the CD4+ T cell subsets and fibroblasts or vascular SMCs within EAT using CellphoneDB (Figure 2H). In brief, one pair of ligand-receptor interaction (TNF_TNFRSF1A) between CD4+ Tem1 and fibroblasts, and the interaction of TNFRSF1B_GRN between CD4+ Tem2 and fibroblasts were significantly reduced in the Adults-HF group compared to the Adults-Non HF group. The dot plots showed that the interaction of HLA-C_FAM3 between CD4+ Tn and vascular SMCs, and interactions of BSG_PPIA and LRPAP1_SCRT1 between CD4+ Tem1 or CD4+ Tem2 and vascular SMCs, were significantly enhanced in patients with HF relative to the healthy control. Whereas the interactions of TNF_TNFRSF1A, TNFRSF1B_GRN and CCL5_ACKR1 between CD4+ Tem1 or CD4+ Tem2 and vascular SMCs were significantly weakened in HF patients (Figure 2H).

**Figure 3:**
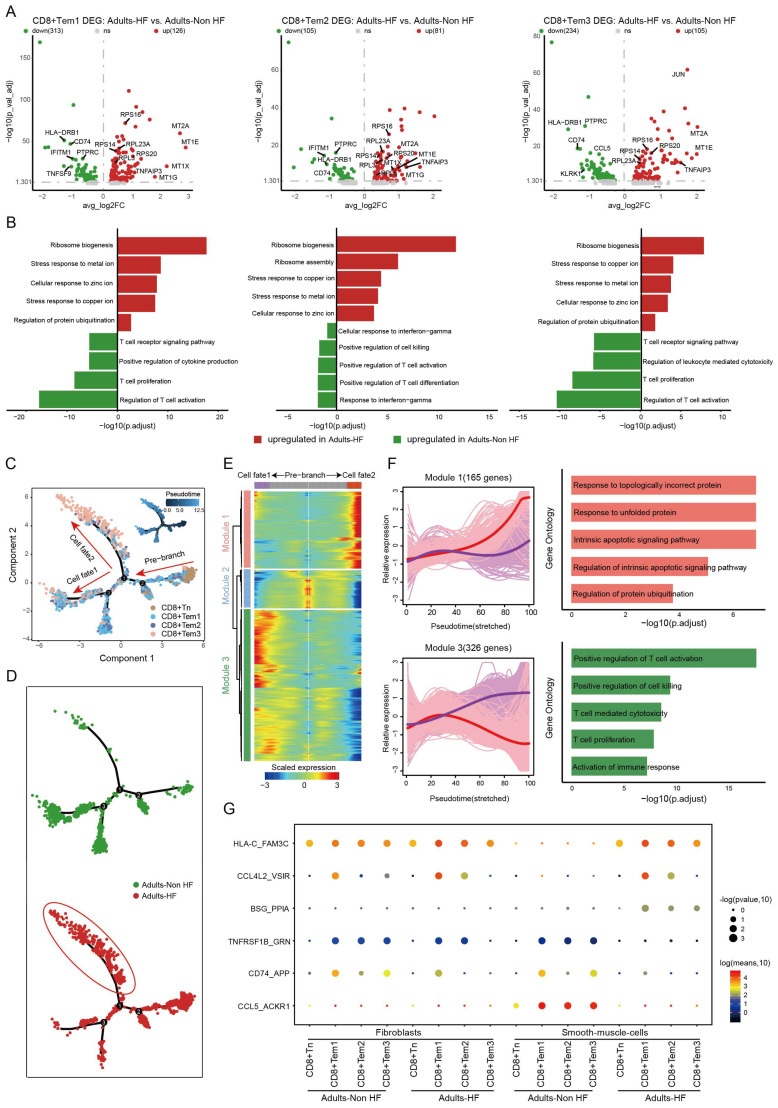
** Functional changes in CD8+ T cells in EAT of HF patients. (A)** Volcano plots illustrating the DEGs of CD8+ Tem1 (left), CD8+ Tem2 (middle), and CD8+ Tem3 (left) cell subsets between the Adults-Non HF and Adults-HF groups. Each dot represents a DEG, with green and red representing down- and up-regulated respectively. **(B)** Bar plots displaying the comparisons of pathway activities of CD8+ Tem1 (left), CD8+ Tem2 (middle), and CD8+ Tem3 (left) cell subsets between the Adults-Non HF and Adults-HF groups. **(C-D)** Pseudo-time trajectories of the CD8+ T cells inferred by Monocle analysis. Cells are colored according to cell type (C) and group (D). **(E)** A heatmap shows the expression patterns of top DEGs that assigned into three major modules in a pseudo-time manner. **(F)** The expression dynamics of genes in modules 1 and 3 under cell fate1 and cell fate 2 states (left), and their enriched biological progresses (right). **(G)** Bubble chart shows selected ligand-receptor interactions between CD8+ T cells and fibroblasts or SMCs.

The expression patterns of CD8+ T cell subpopulations were compared between the Adult HF and Adult Non-HF groups to gain insight into the transcriptomic changes of CD8+ T cells in the EAT during the pathogenic process of HF. Volcanic maps showed 439 DEGs in CD8+ Tem1 cells, 186 DEGs in CD8+ Tem2 cells, and 339 DEGs in CD8+ Tem3 cells, were identified between the two groups (Figure 3A). Genes involved in zinc and copper ion metabolism (eg. genes named *MT1E*, *MT2A*, *MT1X*, *MT1G*) and ribosome synthesis (eg. genes named *RPS16*, *RPL23A*, *RPS20*) were enriched in GO enrichments of these DEGs, whereas the downregulated genes (eg. *IFITM1*, *CD74*, *HLA-DRB1*, etc.) mainly involved in T cell activation (Figure 3B). The differentiation trajectory of CD8+ T cells exhibited a branched structure, starting with CD8+ Tn that differentiated into effector memory T Cells (including CD8+ Tem1, 2, 3; Figure 3C). The pseudo-time trajectories revealed that the CD8+ Tn cells mainly underwent cell fate2 differentiation pathway in patients with HF (Figure 3D). According to the expression patterns along the pseudo-time axis, we grouped the differentially expressed genes in CD8+ T cells into three modules (Figure 3E). Module 1 (165 genes, eg. *ING1*, *AATK*, *UBE2S*, *H3F3B*, *MCL1*) includes genes highly expressed in cell fate2 (red line), which was primarily involved in biological functions such as responses to topologically protein and unfolded protein, intrinsic apoptotic signaling pathway, and the regulation of protein ubiquitination. While genes clustered in Module 3 (326 genes, eg. *ZNF331*,* JUND*, *SNRPG*,* RO60*, *PTGES3*) were responsible for T cell activation and highly expressed under cell fate1 (Figure 3F).

As before, cell-cell communication analysis was performed between CD8+ T cells and fibroblasts or SMCs. Results revealed that the interactions of CD74_APP between CD8+ T cell subtypes and fibroblasts were significantly weakened in patients with HF relative to the healthy control. Meanwhile, interactions of TNFRSF1B_GRN, CD74_APP, CCL5_ACKR1 between CD8+ T cell subtypes and SMCs were significantly weakened, and HLA-C_FAM-3C, CCL4L2_VSIR, BSG_PPIA between CD8+ T cell subtypes and SMCs were significantly enhanced in HF patients (Figure 3G).

### Re-clustering of the myeloid cell subtypes and differential regulation of gene expression in monocyte subsets of HF

**Figure 4:**
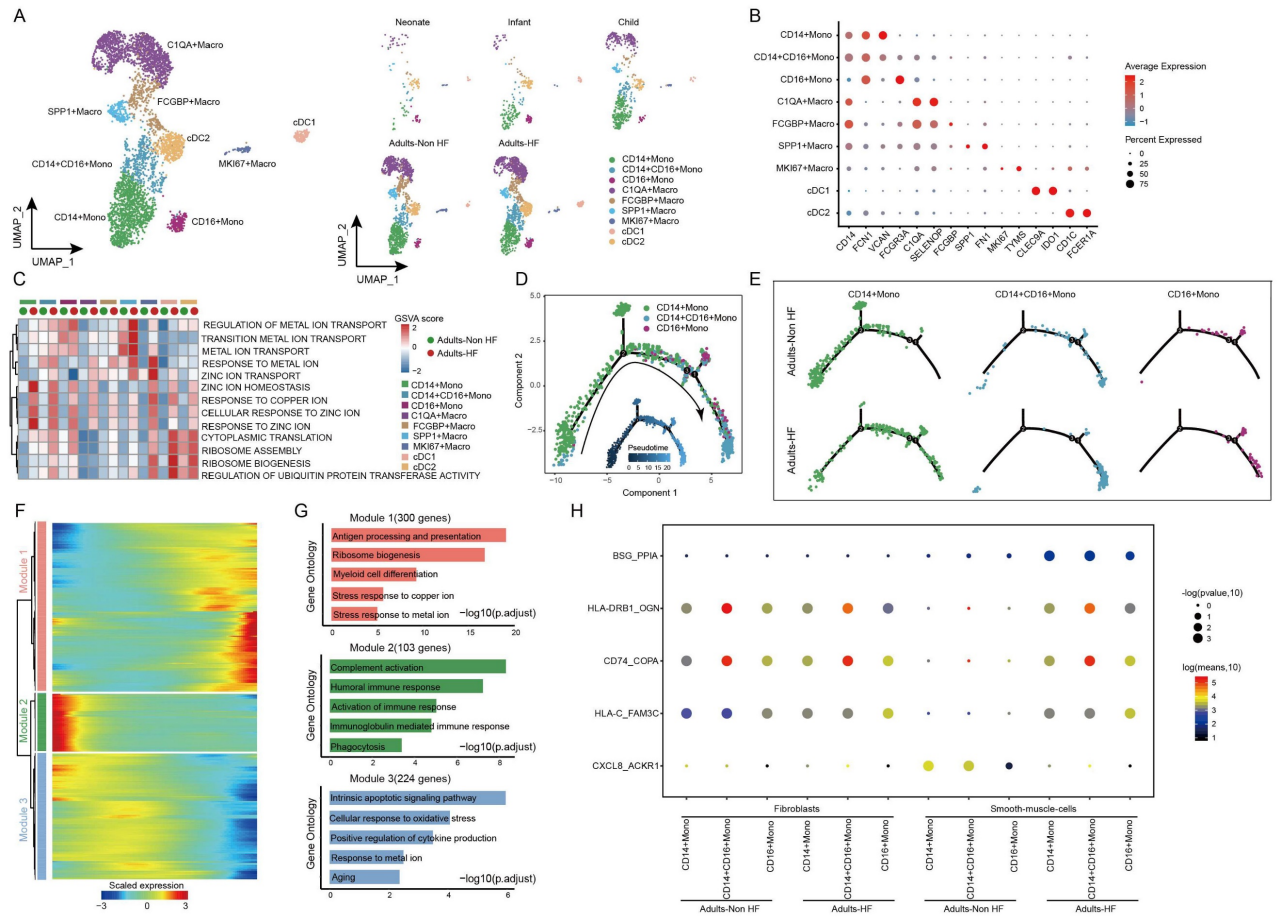
** Diverse subsets were identified in myeloid cells of EAT and functional changes in monocytes of HF patients. (A)** Visualizing subclusters of myeloid cells in human EAT using UMAP plots by cell identity (left) and different groups (right). Each dot represents a cell, colored according to cell type.** (B)** Bubble chart of marker genes for each identified cell subtype in myeloid cells. The expression of a gene is marked by the presence of a cercle, the size of which indicates the proportion of cells that expressing this gene. Color gradient from blue to red indicates a low-high level of expression. **(C)** A heatmap shows comparison of pathway activities across different myeloid cell subtypes in Adults-Non HF and Adults-HF groups by GSVA. The scores of pathways are normalized. **(D)** Pseudo-time trajectory of the monocytes inferred by Monocle analysis. Cells are colored according to cell type. **(E)** The trajectories of CD14+ Mono, CD14+CD16+ Mono, and CD16+Mono in the Adults-Non HF and Adults-HF groups respectively. **(F)** A heatmap shows the expression patterns of top DEGs that assigned into three major modules in a Pseudo-time manner. **(G)** The top biological progresses enriched in each module. **(H)** Bubble chart shows selected ligand-receptor interactions between monocyte subsets and fibroblasts or SMCs.

The 5418 myeloid cells in EAT were clustered into nine distinct groups, including three monocyte subtypes (CD14+ Mono, CD14+CD16+ Mono, and CD16+ Mono), four macrophage subtypes (C1QA+ Macro, FCGBP+ Macro, SPP1+ Macro, and MK167+ Macro) and two classical or tissue-resident dendritic cell subtypes (cDC1 and cDC2) (Figure 4A, Supplementary Table S4). The signature genes of each subset are displayed in Figure 4B. By comparing pathway activities, we further characterized the functions of different myeloid cell subtypes. GSVA results showed that pathways involved in ribosome synthesis (eg. ribosome assembly, ribosome biogenesis, and cytoplasmic translation) and ubiquitination (eg. regulation of ubiquitin protein transferase activity) were significantly upregulated in monocytes, MK167+ Macro and cDC cells of adults HF patients compared to control. Meanwhile, metal ion transport-related pathways were significantly increased in monocytes and macrophages, and responses to zinc and copper ions-related pathways were significantly enhanced in all the myeloid subsets of adult patients with HF (Figure 4C).

Monocle trajectory analysis inferred a differentiation trajectory for monocytes, which started from CD4+ Monos to CD14+CD16+ Monos and culminated in CD16+ Monos as terminal differentiation cluster (Figure 4D). Interestingly, a relatively higher proportions of CD14+CD16+ Monos and CD16+ Monos were observed in Adults-HF group compared to that in Adults-Non HF group at the end of the reprogramming trajectory (Figure 4E). The significant differentially expressed genes along the pseudo-time trajectory were assigned into three modules base on their expression patterns (Figure 4F). Module 1 (300 genes, eg. *MT1G, BLVRB, RPL22, EEF2, RPL7A*) included genes increased their expression levels along the pseudo-time trajectory from the beginning, and preferentially enriched in antigen presentation and processing, ribosome biogenesis, myeloid cell differentiation, stress response to copper ion, and stress response to metal ion. Module 2 (103 genes, *IGLV6-57, IGLV1-51, ERICH1, SFPQ, RB1CC1*) consists of genes whose expression levels declined along the reprogramming trajectory, and were preferentially responsible for immune response (eg. complement activation, humoral immune response, phagocytosis etc.). Genes clustered within Module 3 (224 genes, eg. *ALOX5AP, SOD2, XIST, PNRC1, FOS*) were predominantly associated with intrinsic apoptosis and response to oxidative stress, whose expression levels were upregulated at the beginning and subsequently downregulated along the reprogramming trajectory (Figure 4G).

We further observed five pairs of ligand-receptor interaction differed significantly between adults with HF and healthy control (Figure 4H). The interactions of BSG_PPI, HLA-DRB1_OGN, CD74_COPA, and HLA-C_FAM3C between monocyte subsets and SMCs enhanced their interactions, while CXCL8_ACKR1 weakened their interaction in the the Adults-HF group relative to the Adults-Non HF group.

### Distinct endothelial cell subtypes identified in EAT and the pseudo-time trajectory reconstruction of lymphatic endothelial cell in HF

**Figure 5:**
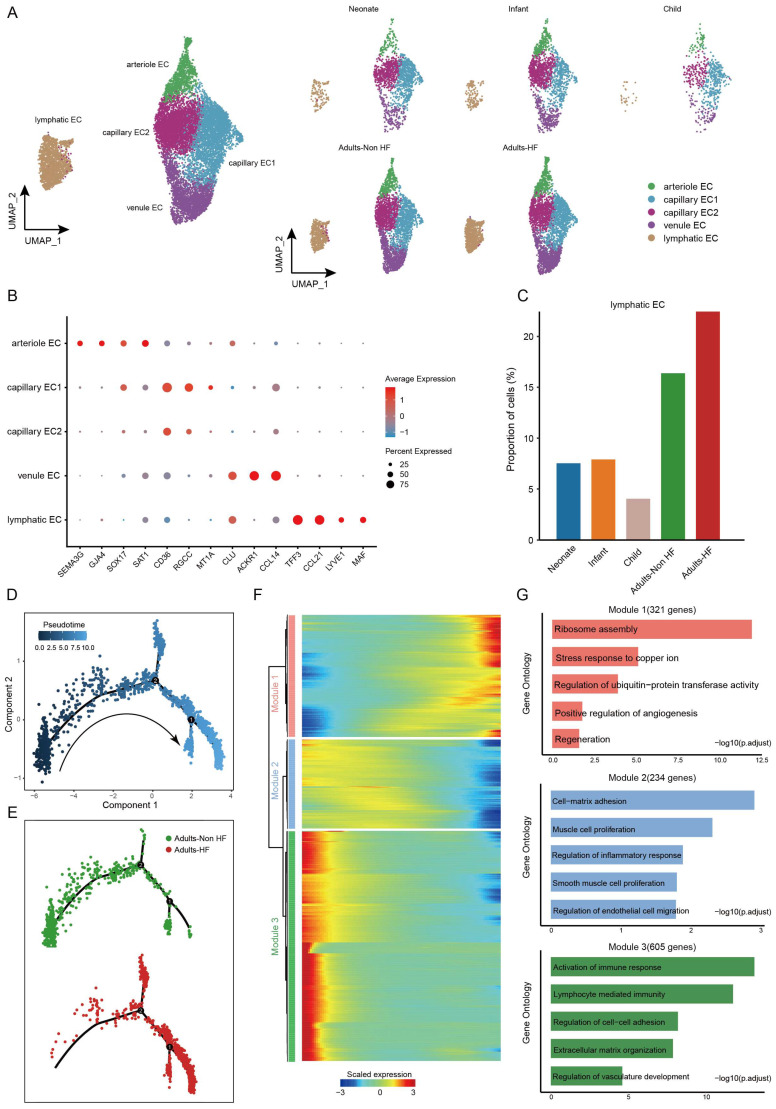
** Endothelial cells clustered into five subsets and their functional changes along the Pseudo-time trajectory in EAT of HF patients. (A)** The visualization of these sub-clusters is depicted through UMAP plots, with each dot representing a cell and its color indicating the cell type. The UMAP plots display the different groups of endothelial cells. **(B)** Bubble chart of marker genes for each identified cell subtype in endothelial cells. The expression of a gene is marked by the presence of a cercle, the size of which indicates the proportion of cells that expressing this gene. Color gradient from blue to red indicates a low-high level of expression. **(C)** Bar plots shows the proportions of lymphatic endothelial cell in different groups. **(D-E)** Pseudo-time trajectory of the endothelial cells inferred by Monocle analysis. Cells are colored based on the predicted Pseudo-time (D) and groups (E). **(F)** A heatmap shows the expression patterns of top DEGs that assigned into three major modules in a Pseudo-time manner. **(G)** The top biological progresses enriched in each module.

A total of 14272 endothelial cells (ECs) were assigned into five subsets according to their differentially expressed markers: arteriole EC, capillary EC1, capillary EC2, venule EC, and lymphatic EC (Figure 5A-B, Supplementary Table S5). The distributions of five subtypes of endothelial cells in each group are shown in Figure 5A right, and marker genes of each subset are displayed in Figure 5B. In HF patients, lymphatic EC proportions were significantly higher than in controls when comparing each cell subtype ratio (Figure 5C), indicating that the lymphatic ECs played a role during the pathogenic process of HF.

Pseudo-time analysis was further performed on lymphatic ECs, and we captured a linear differentiation trajectory that from an initial healthy state to the HF state along the arrow direction (Figure 5D-E). The top genes that significant differentially expressed along the pseudo-time trajectory in lymphatic EC were shown by heat map, which were mainly divided into three modules (Figure 5F). Genes included in Module 1 (321 genes, eg. *ODC1, PFDN5, RPS3, RPLP0, RACK1*) upregulated their expressions from the beginning and expressed high at the end of the pseudo-time trajectory, i.e. genes that were highly expressed in patients with HF, and were predominantly related to ribosome assembly and copper ion metabolism (Figure 5F-G). Genes clustered within Module 2 (234 genes, eg.* SMCHD1, POGZ, PCNX4, SERINC3, CDK13*) were mainly associated with cell-matrix adhesion, muscle cell proliferation, regulation of inflammatory response, SMCs proliferation, and regulation of ECs migration, whose expression levels were upregulated at the beginning and subsequently downregulated along the reprogramming trajectory (Figure 5F-G). Module 3 (605 genes, eg. *TNKS1BP1, LINC00924, RIMKLB, MAP3K20, SMARCA5*) consists of genes that are initially highly expressed and significantly downregulated along the pseudo-time trajectory in HF patients, are preferentially involved in lymphocyte immunity (Figure 5F-G). These results suggested that the reduced normal and immune functions, and the upregulated ribosome synthesis and copper ion metabolism-related functions in ECs of EAT has promoted the progression of HF.

### Fibroblasts subcluster into distinct cell subtypes and the pseudo-time trajectory reconstruction for development

**Figure 6:**
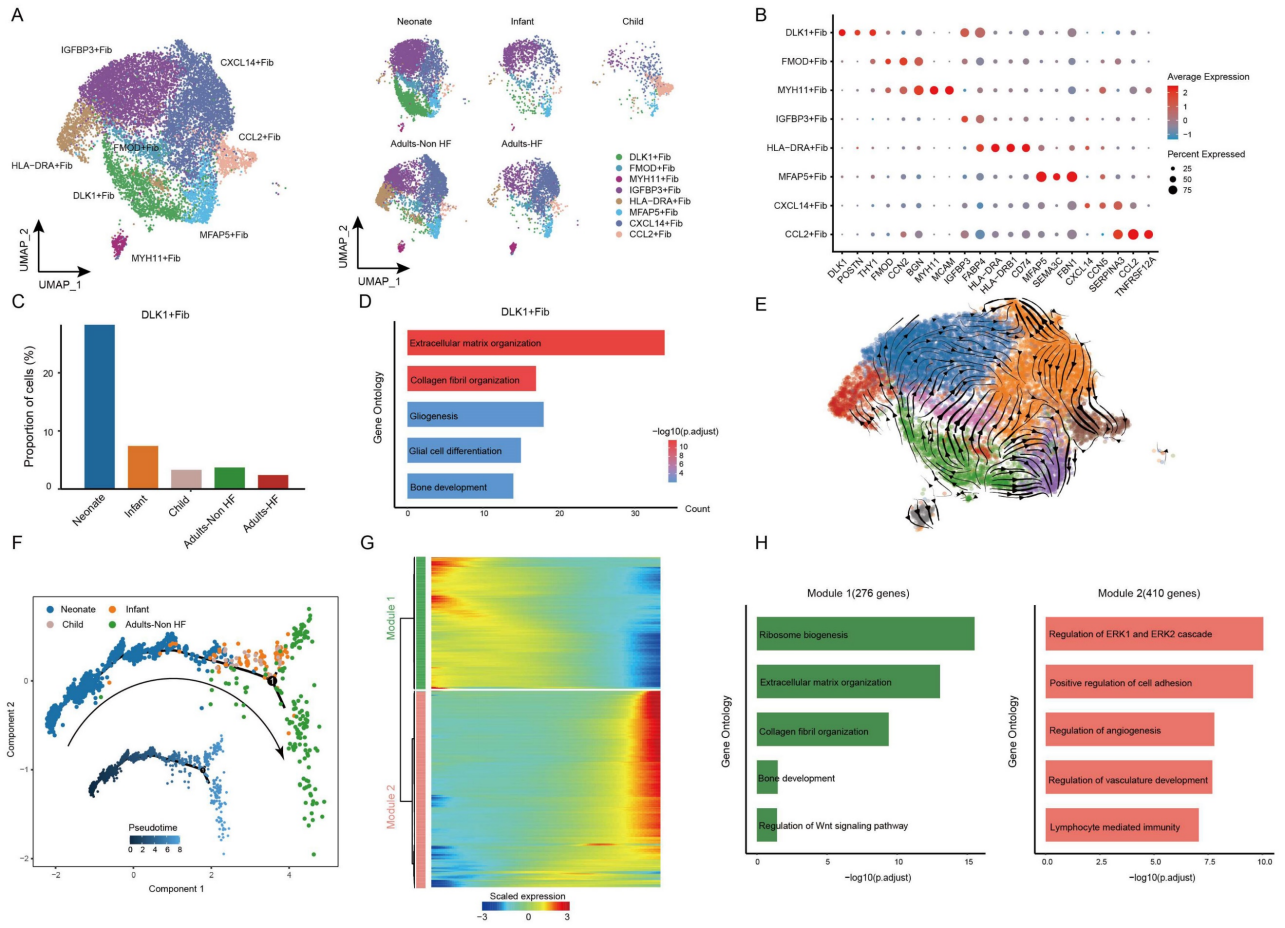
** Fibroblasts clustered into distinct cell subtypes and their functional changes along the Pseudo-time trajectory in EAT of different development stages. (A)** The utilization of UMAP plots to visually represent fibroblast sub-clusters in human EAT, based on cell identity (left) and different groups (right), is demonstrated in this study. Each dot on the plot represents a cell, with color indicating the specific cell type. **(B)** A bubble chart is employed to display the marker genes associated with each identified cell subtype within the fibroblast population. The expression of a gene is marked by the presence of a cercle, the size of which indicates the proportion of cells that expressing this gene. Color gradient from blue to red indicates a low-high level of expression. **(C)** Bar plots shows the proportions of DLK1+ Fib in different groups. **(D)** GO enrichment analysis of marker genes of DLK1+ Fibs. **(E)** UMAP diagram of the RNA velocity of fibroblasts. **(F)** Pseudo-time trajectory of the fibroblasts inferred by Monocle analysis. Cells are colored based on different groups. **(G)** A heatmap shows the expression patterns of top DEGs that assigned into two major modules in a Pseudo-time manner. **(H)** The top biological progresses enriched in each module.

Afterwards, we performed unsupervised clustering of fibroblasts (n = 13121) and observed further heterogeneity between the eight subsets (including DLK+ Fib, FMOD+ Fib, MYH11+ Fib, IGFBP3+ Fib, HLA-DRA+ Fib, MFAP5+ Fib, CXCL14+ Fib, and CCL2+ Fib) (Figure 6A-B, Supplementary Table S6). A specifically high proportion was observed in DLK+ Fibs in neonate compared to other groups (Figure 6C), indicating a strong stemness of this cell subtype. GO enrichment analysis revealed that DLK+ Fibs preferentially expressed genes (eg. *COL1A1, DLK1, MDK, COL3A1, SPARC*) involved in extracellular matrix organization and collagen fibril organization (Figure 6D), and the enriched biological pathways in other fibroblast subsets were displayed in Supplementary Figure S2. Further, results of RNA velocity analysis in fibroblasts of EAT predicted the strongest stemness of DLK+ Fibs (cells in green) with arrows pointing outward, which presenting the direction of cell differentiation (Figure 6E).

The utilization of monocle trajectory analysis was employed in order to reconstruct and delineate the interconnections between various groups (Neonate, Infant, Child, and Adults-Non HF) of fibroblasts, as well as to infer pseudo-time trajectories. As it shown, the fibroblasts appeared to develop in a linear trajectory that along the direction of neonate - infant - child - adult (Figure 6F). In order to more comprehensive insight into the dynamics of gene expression during the transition from neonatal to adult states, we identified two major transcriptional gene clusters based on their distinct expression patterns (Figure 6G).

Module 1, consisting of 276 genes such as *ABCA8*, *SOX4*, *RPS27A*, *RPL18*, and *RPS3A*, exhibited early expression and subsequent downregulation along the reprogramming trajectory. These genes were primarily involved in the regulation of ribosome biosynthesis, extracellular matrix organization, and collagen fibril organization (Figure 6H). Meanwhile, genes enriched in Module 2 (410 genes; eg. *SPRR2F, C5orf38, RSAD2, STK32B, CDKN2A*) were upregulated from the beginning and expressed high at the end of the pseudo-time, and were primarily participate in regulation of cell adhesion (Figure 6H). These results showed functional changes of fibroblasts during their transformation from neonate to adult.

### Diverse SMC subsets identified in EAT and the pseudo-time trajectory reconstruction in HF

**Figure 7:**
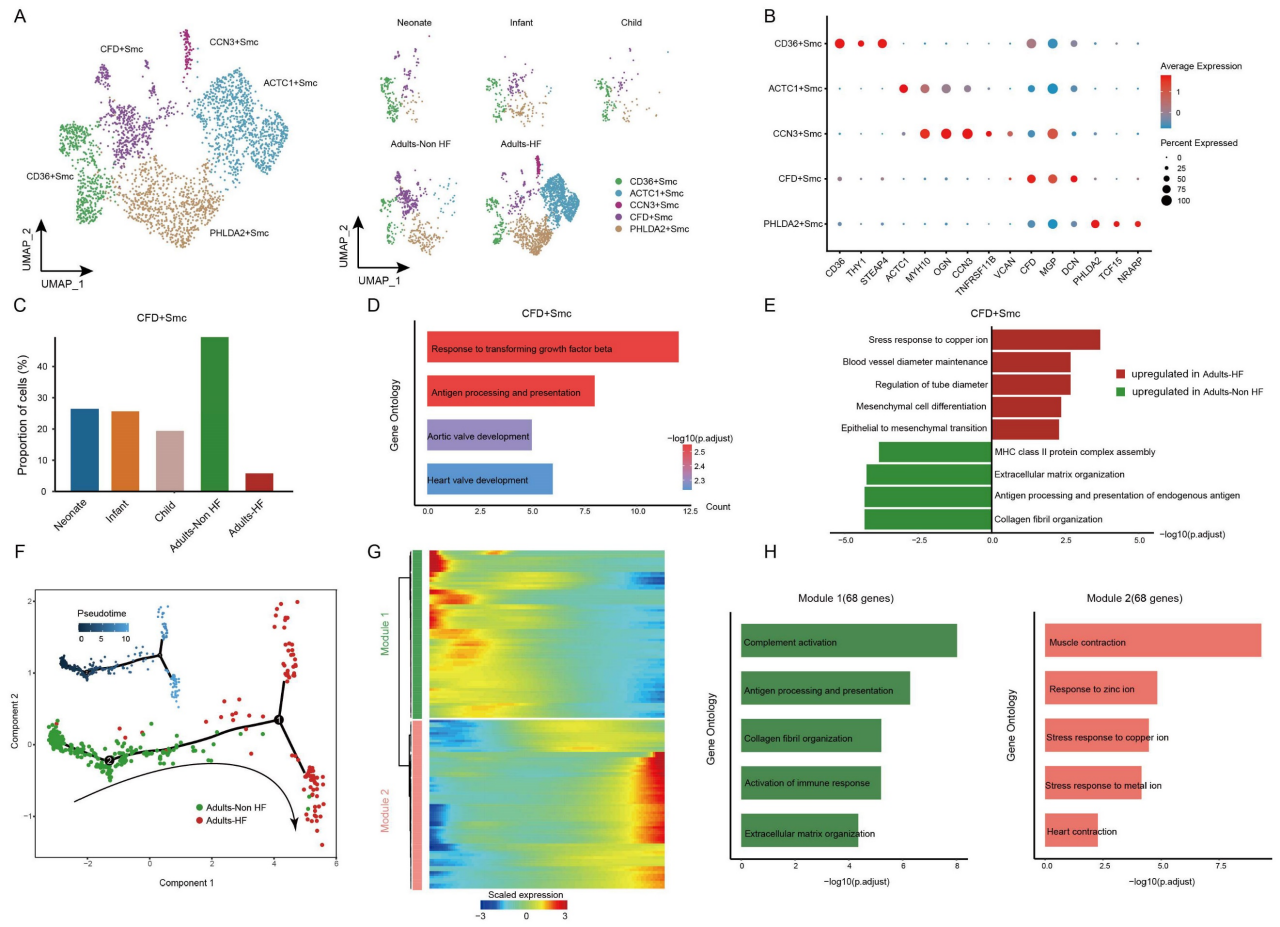
** SMCs clustered into distinct cell subtypes and their functional changes along the Pseudo-time trajectory in EAT of HF patients. (A)** The utilization of UMAP plots to visually represent SMC sub-clusters in human EAT, based on cell identity (left) and different groups (right), is demonstrated in this study. Each dot on the plot represents a cell, with color indicating the specific cell type.** (B)** A bubble chart is employed to display the marker genes associated with each identified cell subtype within the SMCs population. The expression of a gene is marked by the presence of a cercle, the size of which indicates the proportion of cells that expressing this gene. Color gradient from blue to red indicates a low-high level of expression. **(C)** Bar plots shows the proportions of CFD+ SMC in different groups. **(D)** GO enrichment analysis of marker genes of CFD+ SMCs. **(E)** Comparison of pathway activities between the Adults-Non HF and Adults-HF groups of CFD+ SMCs. **(F)** Pseudo-time trajectory of the SMCs inferred by Monocle analysis. Cells are colored based on different groups. **(G)** A heatmap is presented to depict the expression patterns of top DEGs assigned to two major modules, in a pseudo-time manner. **(H)** The top biological progresses enriched in each module.

Finally, all SMCs (n = 3592) in EAT were unsupervised re-clustered and divided into five subsets: CD36+ SMC, ACTC1+ SMC, CCN3+ SMC, CFD+ SMC, and PHLDA2+ SMC (Figure 7A-C, Supplementary Table S7). The distributions of each cell subtype in different groups are shown in Figure 7A right, and the signature genes of each cell subtype are presented in Figure 7B. Upon comparing the proportions of each cell subtype among different groups, a statistically significant reduction was found in the proportion of CFD+ SMC in HF patients compared to the healthy control (Figure 7C), suggesting that the CFD+ SMCs have an influence on the pathogenic process of HF. The GO annotation of preferentially expressed marker genes in CFD+ SMCs (eg. *CCDC144NL-AS10, SUGCT, CCN3, ITGBL1, TNFRSF11B*) showed their capacity to response to transforming growth factor beta and antigen processing and presentation (Figure 7D), while the biological processes that enriched in CD36+ SMC, ACTC1+ SMC, CCN3+ SMC, and PHLDA2+ SMC were shown in Supplementary Figure S3.

We further characterized the functional changes of CFD+ SMCs by comparing pathway activities. The results revealed the disorder of copper ion metabolism for correlated pathway upregulated (such as stress response to copper ion), and weakened immune response in CFD+ SMCs of adult patients with HF (Figure 7E). Next, the pseudo-time trajectory inferred that the direction of differentiation of SMCs from a healthy state to the transcriptomic state of HF patients (Figure 7F). For a better understanding of gene expression dynamics along the pathway, we evaluated two major categories of transcript gene modules on basis of characterized expression patterns (Figure 7G). As reprogramming progressed, genes in Module 1(68 genes; eg. *IGLC2, IGHG1, GLV1-51, ACKR1, IGHG2*) expressed early and their expression decreased, i.e. genes expressed primarily in healthy adults, were largely related to immune response (eg. complement activation, antigen processing and presentation) (Figure 7H). We observed the expression level of genes in Module 2 (68 genes; eg. *SLN, OSR2, ACTG2, RAMP1, ACTC1*) were upregulated from the initial stages of the reprogramming trajectory and highly expressed at the pseudo-time endpoint, i.e. genes mainly expressed in adult patients with HF, were primarily involved in the regulation of muscle contraction and responses to zinc and copper ions (Figure 7H). These results showed functional changes of SMCs during their transformation from a healthy state to the transcriptomic state of HF patients.

## Discussion

EAT is composed of adipocytes, endothelial cells (blood and lymph vessels), nerve cells, immune system cells, and stromal cells^27^. Collectively, these cellular components play crucial roles in tissue-related endocrine organ, energy storage, and energy metabolism, and formed a complex microenvironment due to the complex intercellular communications^28^. In contrast to the visceral adipose tissue situated in the abdomen, EAT exerts primarily localized effects and possesses a distinctive anatomical proximity to the heart, facilitating cellular communication and functional associations between ectopic adipocytes and cardiomyocytes^29^. Its secretion of various immune cytokines may produce exocrine and paracrine effects on myocardium^18^, ^30^. Heart failure is a complicated medical condition with an unfavorable outlook, marked by either diastolic or systolic heart dysfunction^31^. Recent studies have found that EAT as a dynamic immune organ, has a significant impact on the pathogenesis of HF^18^, ^19^. To comprehend the dynamics of EAT cell types in tissue development and the progression of HF, the developmental trajectories of major cell types in EAT were plotted, and cellular immunological features of patients with HF were illustrated using scRNA-seq in this paper, which is a novel technique that enables the explorations of cell trajectories and heterogeneity among different cell types^32^.

The immune activation in the EAT of patients with HF has been noted, specifically the aggregation of T lymphocytes^19^. Vyas et al. (2021) found that the adaptive immune cells showed significant EAT enrichment^33^, ^34^. Based on our analyses, both T and B lymphocytes were highly amplified in EAT of HF patients (Figure 1D). Cell trajectories showed that the CD4+ Tn cells could be activated into either the CD4+ Tem1 cluster or the CD4+ Tem2 cluster (Figure 2D), while the CD8+ Tn cells that differentiated into effector memory T Cells (including CD8+ Tem1, 2, 3; Figure 3C). However, contrary to the results reported by Zhang et al. (2023) [19]that the EAT from HF patients was enriched in immune activation-related pathways, our results showed that the immune response-related pathways (such as T cell activation) were downregulated in major immune cell types (including CD4+ and CD8+ T cells, and monocytes) of EAT from HF patients. This may be due to the later stage of the HF patients we selected, when the immune cells were already activated and the immune function declined. In addition, a relatively higher proportions of CD14+CD16+ Monos and CD16+ Monos were observed in EAT of HF patients (Figure 4E). Prior research indicates that CD16+ monocytes are considered nonclassical-monocytes, and this cell category has conventionally been believed to possess immune-regulatory properties and contribute significantly to metabolic disorders^35^.

In addition to immune system cells, the non-immune cells in EAT also involved in the development of cardiovascular disease^4^. For example, researchers found that ECs are positively involved in both innate and adaptive immune responses except their role in physiological processes. ECs exert their innate immune functions through cytokine secretion, phagocytosis, antigen presentation, etc.^36^.In this paper, we found that the lymphatic EC subset, which downregulated both their physiological functions (eg. cell-matrix adhesion, muscle cell proliferation, SMCs proliferation, and regulation of ECs migration) and immune functions (such as biological progresses involved in regulation of inflammatory response, and lymphocyte immunity), promoted the progression of HF.

In contemporary times, fibroblasts have transcended their conventional role as mere structural constituents of organs, assuming an active role in immune processes. Their pivotal engagement with immune cells primarily occurs through paracrine signaling mediated by the secretion of cytokines and chemokines, as well as the transfer or mobilization of the extracellular matrix microenvironment^37^. In this study, we found the strongest stemness of DLK+ Fib subtype among fibroblasts, which decreased its proportion with age. The functions of fibroblasts were predominantly responsible for regulations of ribosome biosynthesis, extracellular matrix organization, and collagen fibril organization in neonate, while transformed into primarily participate in regulation of cell adhesion in adult (Figure 6H). In addition to the basic physiological functions, fibroblasts have been reported to modulate the behavior of immune cells by producing proinflammatory cytokines, notably tumor necrosis factor and interferon-gamma^37^. The inhibitory communications between fibroblasts and immune cells were observed, such as ligand-receptor interactions of TNF_TNFRSF1A, TNFRSF1B_GRN and CD74_APP between T cell subsets and fibroblasts were reduced, suggesting a decline of immune response of EAT in HF patients. For example, TNFRSF1A (tumor necrosis factor receptor superfamily member 1A) is a target gene of STAT3 that regulates the NF-κB pathway and mediates apoptosis^38^.

VSMC are the main components that constituting the middle layer of the artery. VSMC has been shown to promote the aggregation of immune cells in the outer membrane by forming the arterial third lymphoid organ (ATLO) and upregulating lymphogenic chemokines^39^. Based on our results, we also found a CFD+ SMC subtype, which preferentially responsible for responding to transforming growth factor beta and antigen processing and presentation (Figure 7D), impact the pathogenic process of HF. As mentioned in other major cell types, the immune-related functions (eg. complement activation, antigen processing and presentation) of SMCs in EAT were downregulated during their transformation from a healthy state to the transcriptomic state of HF patients. The inhibitory communications (eg. TNF_TNFRSF1A, TNFRSF1B_GRN, CCL5_ACKR1, CD74_APP, and CXCL8_ACKR1) and enhanced communications (eg. HLA-C_FAM3C, BSG_PPIA, RPAP1_SCRT1, CCL4L2_VSIR, HLA-DRB1_OGN, and CD74_COPA) between SMCs and immune cells were widely observed, showing a disordering immune response to HF in SMCs of EAT. Recent studies have indicated that CCL5 have the potential to act as a crucial effector molecule in the context of T lymphocytes in EAT. This chemokine can be generated by various cell types, including T lymphocytes, macrophages, fibroblasts, and epithelial cells. Its primary function involves regulating the migration of T lymphocytes and monocytes^40^. Another noteworthy factor, FAM3C, plays a significant role in glucose and lipid metabolism regulation. It activates the HSF1-CaM-Akt pathway while repressing the mTOR-SREBP1-FAS pathway, thereby suppressing gluconeogenesis and lipogenesis^41^. Consequently, these ligands or receptors may potentially serve as indicators of high-risk HF, offering clinical value as biomarkers for targeted screening or the development of novel therapeutic approaches. Further investigation is warranted to explore these possibilities.

Interestingly, genes associated with metabolisms of zinc and copper ions (eg. genes named *MT1E*, *MT2A*, *MT1X*, *MT1G*) were significantly increased their expression levels in almost all the major cell types (including immune cells of T cells, monocytes, macrophages and cDCs, and non-immune cells of lymphatic EC and SMCs), suggesting that the metabolism of zinc and copper ion was disturbed in EAT from adult patients with HF. Both zinc and copper are essential trace elements for human body, and play important roles in diverse biological processes. For example, Zinc ions could regulate the metabolism of glucose via a variety of pathways under physiological conditions^42^. In a seminal study by Wang et al. (2015), the identification of small molecules capable of inhibiting the copper-trafficking proteins Atox1 and CCS was reported. The consequential disruption of copper trafficking pathways resulted in the induction of cellular oxidative stress and a subsequent reduction in cellular ATP levels. These findings were instrumental in elucidating the underlying mechanisms behind the inhibition of cancer cell proliferation^43^. William et al. (2010) made a significant discovery regarding the methylation of Metallothionein 1E (MT1E) in malignant melanoma. The over-expression of MT1E was found to enhance sensitivity to cisplatin-induced apoptosis, thereby suggesting its potential as a tumor suppressor gene. These findings shed light on the role of MT1E as a potential therapeutic target for the treatment of cancer^44^. Taken together, the identification of these critical DEGs holds great promise as potential therapeutic markers for targeted screening of HF. Further research in this area is warranted to fully comprehend the implications of these DEGs in the context of HF and to explore their therapeutic potential.

## Conclusion

The scRNA-seq datasets covered EAT cells from 1 neonate, 1 infant, 1 child, 2 adults-HF and 2 adults-Non HF in this paper. The cell trajectories of major cell types in EAT were plotted by pseudo-temporal reconstruction, and immune characteristics of metabolism disorder of zinc and copper ions, and downregulated immune response have been described in major cell types of EAT in adult's patients with HF. The present study's identification of cellular and molecular abnormalities in EAT offers valuable insights into the immune pathogenesis of HF and holds potential for informing the development of efficacious therapeutics. Nevertheless, it is crucial to acknowledge the study's limitations, including the scarcity of EAT samples obtained from neonates, infants, children, and adults both with and without HF. This scarcity restricts our capacity to evaluate transcriptome heterogeneity across various developmental stages and in HF patients. Moreover, the scRNA-Seq data sets were mainly obtained for cell types of EAT except adipocytes, due to the technical limitation in preparation of single-cell suspension of adipocytes^45^.

## Supplementary Material

Supplementary figures and tables.
