# Supplementary material for: Single-cell transcriptomics reveals zinc and copper ions homeostasis in epicardial adipose tissue of heart failure
Source: Int J Biol Sci. 2023 Jul 31;19(13):4036–51. doi: 10.7150/ijbs.82844 (PMC10496493; doi:10.7150/ijbs.82844)
Supplement: Supplementary file 1 — Supplementary figures and tables. [file ijbsv19p4036s1.zip › Supplementary materials.pdf]

Supplementary materials

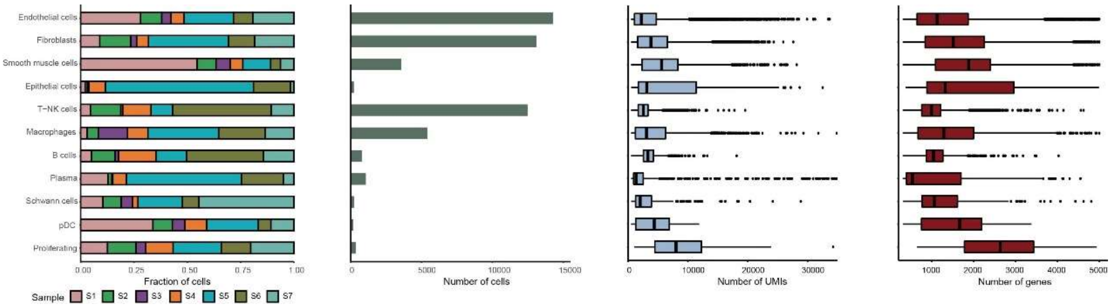

Figure S1: The bar plots of fraction of cells and number of cells, and box plots of the number of UMIs and genes (with the box plot center, box, whiskers, and points corresponding to the median, interquartile range, 1.5× interquartile range, and outliers, respectively) of each cell type in each sample of EAT. S1-S3 were samples from neonate, infant, and child. S4-S5, and S6-S7 were samples from the adults-non HF group and the adults-HF group respectively.

A

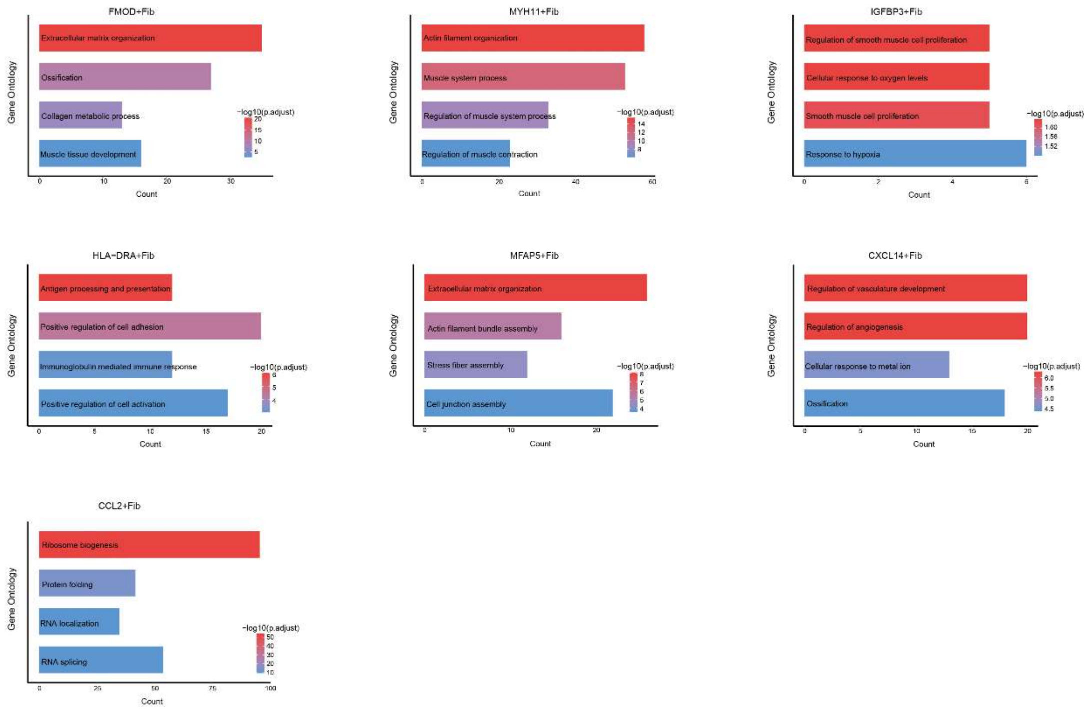

Figure S2: The top enriched biological progresses in fibroblast subclusters (including FMOD+ Fib, MYH11+ Fib, IGFBP3+ Fib, HLA-DRA+ Fib, MFAP5+ Fib, CXCL14+ Fib, and CCL2+ Fib) of EAT.

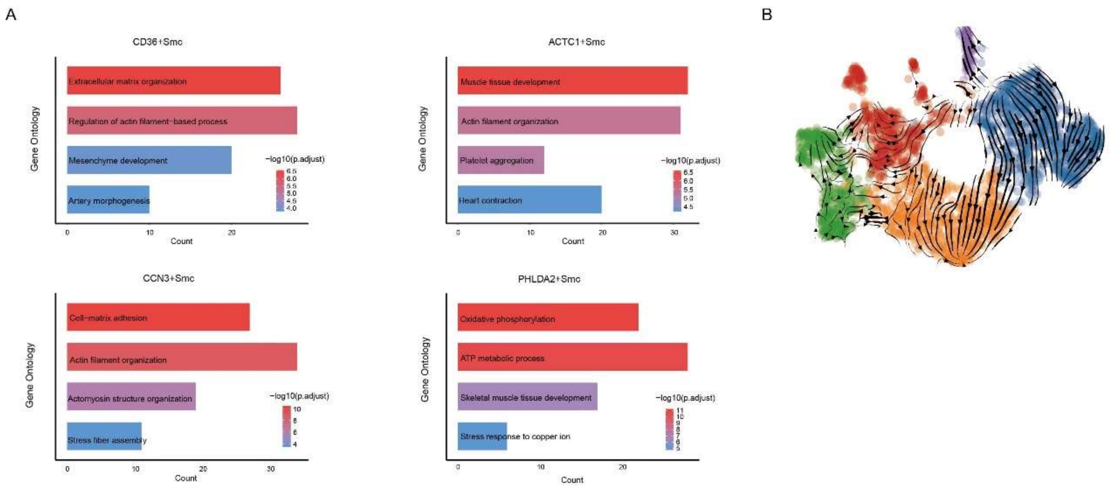

Figure S3: The top enriched biological progresses in SMC subclusters (including CD36+ SMC, ACTC1+ SMC, CCN3+ SMC, and PHLDA2+ SMC) of EAT.

Table S1: The clinical data of the samples from seven participants and their grouping information in this study.

Individual Accession	Individual Name	Sample Accession	Sample Name	Sample Description
HRI667330	NE	HRS878600	S_NE	Neonate
HRI667331	IN	HRS878601	S_IN	Infant
HRI667332	CH	HRS878602	S_CH	Child
HRI667333	HF1	HRS878603	S_HF1	Heart failure 1
HRI667334	HF2	HRS878604	S_HF2	Heart failure 2
HRI667335	NC1	HRS878605	S_NC1	Negative control 1
HRI667336	NC2	HRS878606	S_NC2	Negative control 2

Table S2: Marker genes for distinct cell clusters in EAT.

	Endothelial cells	Fibroblasts	Smooth muscle cells	Epithelial cells	T-NK cells	Myeloid cells	B cells	Plasma	Schwann cells	Prolifering
PECAM1	1.372094	0.087125	0.222126	0.099116	0.106005	0.411654	0.137637	0.361593	0.133521	0.838482
VWF	1.847452	0.168059	0.317637	0.087995	0.116888	0.2196	0.11828	0.218533	0.215265	1.181253
CLDN5	2.241928	0.17926	0.379677	0.111908	0.158031	0.195472	0.16703	0.203271	0.287821	1.209123
CDH5	0.763791	0.032155	0.09773	0.036835	0.026904	0.049147	0.034296	0.037066	0.034349	0.384694
DCN	0.84034	4.458222	1.005999	2.0478	0.694203	1.01717	0.693388	1.268797	1.332498	2.860514
COL1A1	0.307229	2.830999	0.6296	1.137327	0.167077	0.297728	0.186792	0.497207	1.696974	1.847455
DPT	0.20625	2.404786	0.198342	0.260944	0.121247	0.221921	0.138678	0.248268	0.768274	1.55328
C7	0.3893	2.690021	0.463526	0.555387	0.20108	0.387276	0.172401	0.602764	0.807495	1.788695
ACTA2	0.250259	0.379693	3.379636	0.436307	0.179641	0.20203	0.136344	0.114769	0.197548	0.353475
RGS5	0.122964	0.089218	1.451326	0.061324	0.029975	0.094146	0.031796	0.074167	0.1015	0.169123
TAGLN	0.590912	1.022022	4.026312	0.635302	0.276625	0.383064	0.236632	0.289652	0.321466	0.784973
MCAM	0.347624	0.04715	1.488671	0.090517	0.05285	0.046127	0.025322	0.063207	0.320461	0.294554
KRT19	0.007619	0.011568	0.004955	2.671946	0.0087	0.00593	0.007397	0.008214	0.008337	0.008252
KRT8	0.041685	0.015693	0.077209	1.270197	0.005577	0.010001	0.008009	0.011631	0.021628	0.013505
CLDN1	0.001125	0.010544	0.00268	0.806943	0.002813	0.004623	0.002441	0.000162	0.003619	0.002367
CD3E	0.052323	0.054719	0.047079	0.089256	1.441164	0.080914	0.077553	0.102192	0.086706	0.272597
NKG7	0.12978	0.140345	0.117016	0.184849	1.438268	0.185051	0.132707	0.284181	0.142355	0.475079
CD8A	0.014945	0.0123	0.015163	0.016142	0.39982	0.022742	0.020372	0.010281	0.024214	0.105606
CD4	0.014203	0.046987	0.058542	0.011333	0.218063	0.554459	0.027012	0.017543	0.066482	0.072437
LYZ	0.157016	0.176616	0.129875	0.363376	0.086561	2.537922	0.072615	0.688016	0.049891	0.371412
TYROBP	0.120576	0.126214	0.101405	0.15001	0.391465	2.482406	0.088251	0.353969	0.082331	0.261461
FCER1G	0.104331	0.110209	0.100542	0.152469	0.238295	2.312517	0.07447	0.276175	0.134532	0.281128
CD68	0.044273	0.145747	0.040757	0.073199	0.023416	1.125823	0.038747	0.119156	0.075279	0.1256
CD79A	0.007788	0.007985	0.005443	0.012258	0.010241	0.014607	2.502958	0.503911	0	0.009225
CD19	0.00124	0.001939	0.001452	0.004546	0.000243	0.002888	0.341792	0.021843	0.002971	0.004581
MS4A1	0.007576	0.005157	0.007786	0.019494	0.10575	0.010129	1.721401	0.017393	0	0.019109
IGHD	0.001828	0.002445	0.001422	0.006778	0.001801	0.00479	1.242475	0.058512	0	0.001721
MZB1	0.013823	0.009504	0.01561	0.021314	0.025766	0.059849	0.296042	1.878299	0.009791	0.009717
JCHAIN	0.06021	0.047305	0.0439	0.048585	0.085623	0.178971	0.725449	1.863931	0.045369	0.036575
IGHG1	0.12655	0.075258	0.075338	0.306155	0.204206	0.184746	0.432026	2.654909	0.011751	0.093355
MPZ	0.004837	0.004698	0.010281	0	0.005041	0.005789	0.006365	0.001651	1.410123	0.008817
S100B	0.016528	0.156276	0.015483	0.003018	0.050925	0.058844	0.014436	0.009062	2.419218	0.140952
MKI67	0.001648	0.002435	0.001557	0	0.003005	0.006997	0	0.003483	0.013613	0.544429
TOP2A	0.006001	0.007672	0.006819	0.003777	0.004103	0.014309	0.003762	0.004849	0.020752	0.664474

Table S3: Marker genes for distinct cell subsets of T-NK cells in EAT.

Table S4: Marker genes for distinct cell subsets of myeloid cells in EAT.

Table S5: Marker genes for distinct cell subsets of endothelial cells in EAT.

Table S6: Marker genes for distinct cell subsets of fibroblasts in EAT.

Table S7: Marker genes for distinct cell subsets of SMCs in EAT.
